# Experiencing a significant win and its sociodemographic and motivational predictors: A comparative analysis of pure-chance gamblers from Poland and France

**DOI:** 10.1371/journal.pone.0277972

**Published:** 2022-11-21

**Authors:** Bernadeta Lelonek-Kuleta, Rafał P. Bartczuk, Marie-Line Tovar, Emmanuel Benoit, Jean-Michel Costes

**Affiliations:** 1 The Institute of Psychology, The John Paul II Catholic University of Lublin, Lublin, Poland; 2 Pôle d’Innovation et d’Expérimentation sur le Jeu Excessif, Société d’Entraide et d’Action Psychologique, Dijon, France; 3 Associated Researcher, Research Chair on Gambling, Concordia University, Montreal, Canada; University of Nottingham, UNITED KINGDOM

## Abstract

Research on the relationship between wins and gambling behavior often focuses on winning considerably large amounts of money. It seems, however, that it is not the amount of the win, but the significance that the player assigns to the win, that exerts a decisive influence on gambling behavior. Therefore, in this study we adopted the concept of *significant win*: a win perceived by gamblers as important to them. The research aimed to discover what kind of wins are experienced as significant and what factors explain experiencing wins as significant. This study, conducted in Poland (*N* = 3,143) and France (*N* = 5,692), also had a comparative goal: discovering intercultural differences in experiencing significant wins. A computer-assisted web survey was administered to gamblers who engaged in pure-chance gambling, where the participant does not influence the outcome of the gamble after the initial bet is placed—selected from representative samples in both countries. We used logistic regression models to examine predictors of significant win experience in both countries and the differences between the countries. The results demonstrated that Polish gamblers more frequently considered a win significant when it was accompanied by strong, often negative emotions and was higher in monetary value normalized in terms of average monthly personal income, than French gamblers. French gamblers more frequently associated a significant win with a positive experience. The common predictors of a significant win experience in both countries were: being in debt, experiencing the win of a close person, gambling in a game of pure chance other than lotteries, more systematic pursuit of gambling, self-enhancement motivation, and coping motivation to gamble. Age at initiation into gambling was a significant predictor only in the French sample, whereas a financial motivation was a significant predictor in the Polish one. The results confirmed that the subjective perception of gambling wins is only partially related to the amounts of wins, which has practical implications for planning prevention strategies.

## Introduction

Winning at gambling seems to be the main incentive to gamble, and gambling is undoubtedly associated with money. According to some authors, a financial reward, or the hope of it, is the main factor that makes gambling attractive [1, 2]. The importance of financial winning in gambling has been discussed for years. At the same time, there has been a noticeable difficulty in precisely defining the framework of winning, specifically, the point at which it begins to impact gambling involvement significantly. However, it is noticeable that the gamblers individually experience winnings, and the gamblers who have won a small amount may consider themselves a winner [3, 4]. Nevertheless, the scientific literature has focused mainly on the so-called “big win,” which is defined differently depending on the author. The press devotes attention to these so-called big winners (most often, lottery amateurs), and private companies often offer them support in managing their winnings. In Poland, state-owned gambling operator Totalizator Sportowy keeps records of paid winnings of at least PLN2,280 (~ €550); however, the winner’s personal information is protected [5]. Winners’ personal information is also protected in France [6].

Research on winning has focused on several main issues. One is the influence of winning on various aspects of the winner’s quality of life and their environment; another is the association of winning with gambling-related harm and problem gambling; the latter is the relationship between cognitive distortions and the experience of winning.

The results regarding the impact of winning gambling on the winner’s quality of life are inconclusive. Researchers have pointed to an increase in the quality of life after a significant win [7, 8] as well as to the lack of this effect [9]. Winners often keep their jobs, especially when the job is important and satisfying to them; some limit their hours or take longer unpaid leave; unskilled people are significantly more likely to leave their job [10–13]. Very interesting conclusions can be drawn from the research of Agarwal et al. [14]. They showed that a high win by a resident in a neighborhood was associated with an increase in their neighbors’ finances in the following years after this event (something that may result, among causes, from increased consumption or taking out loans). The experience of someone else’s victory is a significant factor that intensifies the willingness of witnesses to gamble [15]. Furthermore, observing a third party winning in laboratory conditions led participants to take more risks when placing bets [16]. Martinez et al. [17] showed that it is not just watching a win, but the belief that a winner was influential in achieving the win, that increases risk taking in gambling.

Neurobiological research has significantly contributed to understanding the relationship between the win–lose experience and gambling addiction. For example, Hollander et al. [18] showed that people experienced increased limbic and sensory activation in response to a blackjack game with a cash prize compared with a game with a token point reward. Experimental studies conducted by Dixon et al. [19] showed that, among pathological gamblers playing a random machine simulator, there was a neuronal activation in the dopamine pathway in response to a significant win but not in response to small wins, which also confirms the existence of a “dose effect” with gambling analogous to that of medicinal substances. In addition, a winning situation causes a significant increase in skin conductivity, the effect of which is absent in a losing situation [20]. Lole and Gonsalvez [21] described the significant phenomenon of reduced sensitivity to winning by problem gamblers. In light of the previous research [22], nonproblematic gamblers show more substantial skin conductivity in response to significant wins than to lesser wins and losses. In contrast, among problem gamblers, there were no significant differences between short-term reactions to big and small wins. The authors of that study even suggested the existence of a potential biological marker of problem gambling. non-problematic.

Research has shown an association between winning and increased risk-taking, a risk factor for problem gambling [23, 24]. However, the relationship between the experience of a significant win and problem gambling is unclear. In light of a recent meta-analysis, the experience of the significant win at the beginning of the play is not significantly related to problem gambling in the later period [25]. Most studies support this relationship [26–28].

Another interesting issue is the estimation of winnings and money spent on a game. Gamblers tend to underestimate their gambling expenditure, a phenomenon that mainly affects problem gamblers [29]. However, distortions also affect winnings and involve overestimating one’s winnings [30]. An important conclusion comes from Kassam et al.’s [31] research, which showed that winners who used scratch cards derive satisfaction from the idea of winning, regardless of the amount. In contrast, losers react to the idea of a loss and its amount. These results suggest that the risk factor that induces people to continue gambling may be the idea of winning, regardless of the amount. In light of Ginley et al.’s [32] study, it is interesting that losing and winning gamblers react differently to the warning messages displayed as they play a random slot machine. Although winning gamblers play less in response to these messages, the effect is very weak in the case of the losing gamblers.

Another factor associated with the experience of winning and losing is cognitive distortions. Research conducted by Dong et al. [33] indicates that a gambler maintains a distorted belief after a losing streak. In contrast, after a winning streak, their tendency to make decisions based on erroneous beliefs, such as that they have an increased chance of winning after experiencing a win, intensifies. Research by Xu and Harvey [34], based on an analysis of more than 500,000 bets made on sporting competitions, showed an increased tendency for gamblers to fall into the “gambler’s fallacy” trap, that is, believing that luck will “reverse.” Therefore, after winning, they make safer bets and, after losing, make riskier ones. In turn, Monaghan et al. [35], in research conducted among students, showed that the tendency to make cognitive errors was significantly weaker in the case of students who lost than in those who won. Verbruggen et al. [36] reported that the experience of a loss, in the context of a potential win, leads to impulsivity. Impulsivity, in turn, has been identified by researchers as a risk factor for problem gambling [27].

According to Young et al. [37], reactions to the results of a game differ depending on the nature of the game. For example, recreational gamblers who play online slots have a decreased desire to play after they experience a significant win or a series of small wins, whereas among problematic gamblers, the desire to play after experiencing a significant win intensifies. Kassinove and Schare [3], on the other hand, demonstrated no differences concerning continued play between gamblers who experienced a significant win and those who experienced a series of losses. An essential aspect of the impact of a win on a gambler is the moment of occurrence. Weatherly et al. [4] showed that gamblers who experienced a significant win in the first round of a game dropped out of the game earlier than those who experienced a win in the fifth round. However, Mentzoni et al. [38] demonstrated in laboratory studies that there were no significant differences in the severity of game craving between nonproblematic gamblers who experienced a significant win under laboratory conditions at the beginning and end of the game session.

An analysis of the literature shows the ambiguity of the terms “significant” and “big win” and the implications of their relationship with the nature of subsequent gambling. Numerous studies have been carried out in laboratory conditions, and studies conducted in the field have usually been concerned with selected aspects of this phenomenon, such as the impact of winning on the choice of health care, neighborly life, professional career, and life satisfaction. Depending on the study, winnings defined as significant/large have included various amounts, from $1.60 [4], $10 [3], and $50 [38], in laboratory studies, up to more than CAD29,000 [14] or $50,000 [12], in studies primarily involving lottery gamblers.

### The present study

In this study, we conducted a comparative analysis between pure chance gamblers from Poland and France to define the concept of a “significant win” and the psychosocial factors that coexist with the experience of a significant win. Because gamblers individually define significant wins, their understanding and experience may be influenced by various factors, including socioeconomic status and cultural context. Therefore, we carried out an international study in two countries with different gambling regulations as well as a diversified socioeconomic context (e.g., the average household income in Poland in 2020 was €8,000, and in France, it was €27,000, according to Eurostat [39]).

Because some studies also have proven the influence of small wins on the intensified willingness to continue gambling [4], we concluded that, in order to get a complete picture of the phenomenon—that is, the importance of winning to the gambler—we could not limit ourselves to very high amounts (i.e., the concept of “big win” that appears in the literature). Considering the research results, we adopted the concept of a “significant win,” as any critical win for the reasons indicated by the winner themself. In addition, bearing in mind the nature of the research that considers winning, we decided to look at this phenomenon from a broader perspective by taking into account various factors related to winning, including the type of gambling, the intensity of gambling, the gambler’s life context, experiences after winning, changes related to the nature of and beliefs about gambling after winning, the definition of a significant win as well as using a reward, experiencing another person’s winning, and psychological factors. Gambling and the probability of winning are perceived differently by gamblers and are influenced, among other things, by the type of gambling. There is a distinction between pure chance games and games of chance and skill [40]. To increase the validity of our results and the likelihood that they will have good generalizability, we decided to include only pure chance gambling, for which there are no additional factors that could objectively affect the result of the game: lotteries, scratch cards, slot machines, roulette, and casino games that are based only on chance. The representativeness of pure choice gambling justifies our choice to use it in the different samples of all French and Polish gamblers (9 out of 10 gamblers say they have played scratch cards, draws, and/or slot machines in the past 12 months [41, 42] and because the share of chance in gambling requiring skill is not comparable (poker, sports betting, etc.) [43–45].

The gambling offer of legal pure chance games in Poland includes lotteries (land-based and online from 2019), scratch cards (at land-based locations and online), slot machines (in land-based slot machines, land-based casinos, and the only legal online casino), and roulette (in land-based casinos and an online casino). All these games, except for scratch cards, are subject to state regulations [46]. Private operators (19 in 2021) can only organize bookmakers in Poland (land-based and online). In France, the gambling options of legal pure chance games include lotteries and scratch cards (land-based and online from 2010) and scratch cards and slot machines in land-based casinos only.

## Materials and methods

The online surveys were conducted in parallel fashion in France and Poland among adults (age 18–64) by companies by means of research panels, with anonymity and the same methodology in both countries ensured. The typical stratified sample selection of the first research stage considered gender, age, and hometown size.

### Participants

The Polish sample comprised 3,143 pure chance case gamblers, selected from a representative sample of 7,320 adult internet users aged 18 to 64.

The French sample was composed of 5,692 pure chance case gamblers, selected using the quota method from a representative sample of 10,004 people aged 18 to 64, representative of the general population.

To be sure we included in the sample of pure chance case gamblers, two questions were asked: “During the past twelve months, what games have you gambled by wagering money, online or at a point of sale?” and “Among the gambling games mentioned by you, which one did you spend the most time or money on?” The sample included people who selected one answer from among the following choices: lotteries; scratch cards; slot machines; and other casino games, except poker.

The following sociodemographic variables were included in the study: gender, age, place of residence, education level, marital status, monthly income, and presence of debts. Table 1 presents the exact characteristics of the respondents in terms of sociodemographic variables. The two samples differed on some of the sociodemographic variables. The statistical significance of these differences was tested, and the results are summarized in Table 1.

**Table 1:** Sociodemographic characteristics of French (*n* = 5,692) and Polish (*n* = 3,143) sample.

Variable	Country				Test	
	FR		PL			
	n	%	n	%	df	χ2
**Place of residence**					4	500.79***
** Rural communes**	1,158	20.34	1,030	32.77^†^		
** 2,000–20,000 inhabitants**	880	15.46^†^	429	13.65		
** 20,000–100,000 inhabitants**	793	13.93	722	22.97^†^		
** >100,000 inhabitants**	1,897	33.33^†^	811	25.80		
** Capital agglomeration**	964	16.94^†^	151	4.80		
**Gender**					1	4.48*
** Male**	2,538	44.59	1,475	46.93^†^		
** Female**	3,154	55.41^†^	1,668	53.07		
**Vocational activity**					1	0.72
** Present**	4,092	71.89	2,286	72.73		
** None**	1,600	28.11	857	27.27		
**Education level**					6	431.92***
** Primary**	146	2.57	84	2.67		
** Basic vocational**	1,012	17.78^†^	357	11.36		
** Secondary with secondary school certificate (SSC)**	1,500	26.35	946	30.10^†^		
** SSC + postsecondary school**	1,302	22.87^†^	407	12.95		
** Undergraduate**	753	13.23^†^	319	10.15		
** Master’s degree/engineering**	895	15.72	1,000	31.82^†^		
** Doctorate**	84	1.48^†^	30	0.95		
**Relationship status**					3	135.22***
** Single**	1,488	26.14^†^	533	16.96		
** Divorced/widowed/separated**	473	8.31^†^	192	6.11		
** Married**	2,776	48.77	1,713	54.50^†^		
** Cohabitating**	955	16.78	705	22.43^†^		
**Children**					2	103.99***
** None**	1,454	34.59	1,351	42.98^†^		
** 1–2**	2,186	52.00	1,572	50.02		
** 3+**	564	13.42^†^	220	7.00		
**Debts**					2	84.32***
** None**	2,703	47.49	1,813	57.68^†^		
** In the past**	2,183	38.35^†^	977	31.08		
** Present**	806	14.16^†^	353	11.23		
**Household income**						
** <€500/month // <PLN 2,000/month**	162	2.80	250	8.00		
** €500–999/month // PLN2,000– 3,999/month**	286	5.00	702	22.30		
** €1,000–€ 1,499/month // PLN4,000–5,999/month**	731	12.80	773	24.60		
** €1,500–2,249/month // PLS6,000–9,999/month**	1,210	21.30	643	20.50		
** €2,250–3,000/month // PLN10,000–11,999/month**	1,212	21.30	111	3.50		
** €3,001–4,500/month // PLN12,000–7,999/month**	1,396	24.50	70	2.20		
** €4,501–6,000/month // PLN18,000–23,999/month**	475	8.30	19	0.60		
** >€6,000/month // >PLN 24,000/month**	182	3.20	43	1.40		
** No answer**	38	0.70	532	16.90		

*Note*. Table cells with a significantly greater proportion of a given category in one of the countries (according to *p* values adjusted for multiple comparisons with the Benjamini–Hochberg method, *p* < .05) are marked by an obelisk (^†^). Household income was not compared between different currencies.

*p < .05.

***p < .001.

The Polish sample was characterized by a rural population and a population in agglomerations of 20,000 to 100,000 inhabitants or higher. In contrast, in the French sample, agglomerations of 2,000 to 20,000 inhabitants and large agglomerations stood out. The French sample had a higher number of females, and the number of children in the household was higher than in Poland. The French sample included more single people, and Poland had more couples. The French participants seemed more concerned about debt than the Poles.

### Measures

The surveys were conducted through an online questionnaire developed by Marie-Line Tovar and Jean-Michel Costes and validated by a steering committee of the Etude Sur Les Impacts des Gains Marquants (Study on the Impacts of Significant Wins).

The questionnaire consisted of seven modules. Three modules focused on the gambler’s current situation: sociodemographic characteristics, and gambling practice. Three other modules collected information about the presence or absence of a significant event in the history of winning gamblers that resulted from their gambling and, for multiple winners, the description of the first and last significant win. Finally, a final module looked at the impulsive traits of these gamblers.

Sociodemographic and gambling-related variables. The sociodemographic and gambling-related variables were measured using a questionnaire. The questions are presented in Table 2. In addition, we recoded the answers for the logistic regression analysis. Table 2 shows the recoding schemes.

**Table 2:** Sociodemographic and gambling-related variables used in the study–their measurement and transformation.

Variable	Question	Transformation	Categories (code)
**Gender**	What is your gender? (2 categories)	None	**Male (1)**
			Female (2)
**Age**	What is your age? (5 categories)	Dichotomized	**≤34 (1)**
			35+ (2)
**Education level**	What is your highest obtained level of education? (7 categories)	Dichotomized	**≤SSC (1)**
			SSC+ (2)
**Household income**	Considering all household income sources, i.e., net income, family benefits, pensions, and other net income, what is the current level of your household income? (9 categories)	Dichotomized according to mean household income (MHI)	**≤MHI (1)**
			MHI+ (2)
**Debts**	Does your household have difficulties with financial obligations (rent, taxes, loans etc.) or securing expenses for the entire month? (3 categories)	None	**None (1)**
			In the past (2)
			Present (3)
**Age at gambling onset**	How old were you when you played gambling for money for the first time? (open-ended numeric)	Dichotomized	**≤19 (1)**
			20+ (2)
**Significant win in an environment**	Before you started to gamble for money, did you know anyone in your close vicinity (family, friends, partner) who had won a significant gambling win at money? (2 categories)	None	Yes (1)
			**No (2)**
**The preferred pure-chance game (PCG)**	Among the gambling games mentioned by you, to which you devoted the most time or spent the most money? (4 categories)	Dichotomized	**Lottery (1)**
			Other PCG (2)
**Spending on gambling**	How much money did you spend on [selected game] in the last month? (open-ended numeric)	Trichotomized based on quartiles	**1Q (1)**
			2Q & 3Q (2)
			4Q (3)
**Frequency of gambling**	How often do you play [selected game]? (5 categories)	Dichotomized	At least once a week (1)
** **			**Regularly, but less than once a week (2)**

*Note*. The categories used as reference categories in logistic regression are in boldface type. MHI data were taken from Eurostat data [39]. SSC = secondary school certificate. 1Q, 2Q, 3Q, and 4Q represent the 1^st^, 2^nd^, 3^rd^, and 4^th^ quartiles.

Definition of a significant win. An international project team developed a proprietary questionnaire to identify how the respondents defined their first and last significant win. The question was, “To be precise, on account of what do you consider the first/last win significant for you…?” The respondent chose between 12 categories (see Table 3) that were developed based on a literature review and the results of qualitative research on significant wins that had been carried out by the French team earlier [47].

**Table 3:** Comparison of definitions of significant win chosen in both countries.

Definition	France		Poland		χ^2^(1)
	(*N* = 1,824)		(*N* = 1,216)		
	*n*	%	*n*	%	
**Because of its value (quantity)/it was a large amount of money.**	428	23.46	217	17.85	13.78***
**It was a win exceeding my standard of everyday life.**	179	9.81	133	10.94	1.0007
**It was a win that I used entirely for gambling and lost.**	80	4.39	78	6.41	6.09*
**It was a win which I added to my budget.**	739	40.52	390	32.07	22.28***
**This win covered all of my losses/covered some of my losses.**	114	6.25	127	10.44	17.58***
**This win happened after a series of losses/after a major loss.**	66	3.62	108	8.88	37.45***
**Because the starting stake was low**	621	34.05	243	19.98	70.92***
**It was a win on a gaming machine.**	178	9.76	109	8.96	0.54
**This win was not enough to cover my losses.**	110	6.03	114	9.38	11.96**
**It was a win of another player (family/friends/acquaintance).**	95	5.21	66	5.43	0.07
**It happened at a difficult time (problems related to relationships, work, social or financial situation).**	138	7.57	141	11.60	14.21***
**It happened at the right moment (e.g., just before the holiday).**	330	18.09	219	18.01	0.003

*Note*. For players experiencing a winning streak or multiple significant wins, the definition of the last significant win was considered.

*p < .05.

**p < .01.

***p < .001.

The respondents were also asked about the amount of the significant win: “What was the amount of the first/last significant win?” (free-answer formula). Finally, the respondents replied regarding their country’s currency (zloty or euro). Because of the economic differences between Poland and France, we normalized the winnings. We compared them with the average income for 1 week per person in both countries based on Eurostat data [48].

Motivation to gamble. Gambling motivation was measured with the Gambling Motives Questionnaire–Financial (GMQ–F) [49]. The Polish translation of the items (except for the financial dimension) [50] was used, and we translated the financial items. In the French survey, appropriate adaptation [51] was used. The GMQ–F comprises 15 items that the respondent rates on a 4-point response scale (1 = *never or almost never*, 2 = *sometimes*, 3 = *often*, 4 = *almost always*). The tool allows researchers to determine the intensity of the four motives of gambling: (1) financial, (2) social, (3) enhancement, and (4) coping. Because we intended to conduct cross-cultural comparisons, we performed the GMQ–F cross-cultural invariance analysis using confirmatory factor analysis. As a criterion of invariance, we adopted, as suggested by Cheung and Rensvold [52], a decrease of .01 or larger in the comparative fit index (Δ comparative fit index) because of the sensitivity of the χ^2^ test to the sample size. After fitting the original model, measurement invariance was not observed at the level of the factor loadings because of the difference in the loading of Item 8 (to earn money). After we removed this item, the method reached invariance, allowing for intercultural comparisons (see the results of model comparisons in S2 Table). We calculated the results of the Financial Motive subscale without that item. In the present study, Cronbach’s α reliability coefficients for subscales in Poland and France were as follows: enhancement, .87 and .82; social, .86 and .83; coping, .89 and .88; and financial, .84 and .79, respectively.

### Statistical analysis

We used standard statistical procedures to analyze the data set. First, we used descriptive statistics to describe each country’s sociodemographic characteristics and variables of interest. Second, one-variable group comparisons were made using the χ^2^-test (followed by adjusted standardized residuals [*e*] analysis) or a Student *t*-test. Finally, logistic regression models were used to examine predictors of significant win experience in both countries and cross-countries differences. We conducted the analyses using the SPSS statistical package [53]. The data underlying the findings described in the present manuscript were deposited in a public repository (DOI: 10.17605/OSF.IO/256TE).

### Ethics

The study procedures were carried out following the Declaration of Helsinki and the standards of good research practice recommended by the American Psychological Association. The participants were informed about the confidentiality and anonymity of the research and the right to resign from participation. The Polish study was approved by the Ethics Committee of the Institute of Psychology at The John Paul II Catholic University of Lublin (KEBN_35/2020). The French study was approved by the steering committee of the research project, which gives it consensus validity and its realization within the framework of the General Data Protection Regulation and the Commission Nationale de l’Informatique et des Liberté (National Commission on Information Technology and Liberties) standards imposed on the sector of the Institutes of French Studies.

Written informed consent was sought and obtained from all respondents—by ticking an appropriate box. For the French study, an invitation was sent by email to a sample of panelists, explaining the survey project and asking them to log on to a dedicated website, if they agreed. By logging on to this address and completing the questionnaire, they agreed to participate in the project. In Poland, when registering in the Ariadna Panel, respondents accepted the terms and conditions (including agreeing to send them invitations to participate in the research). They accepted the terms and conditions by ticking a box. Hence, they agreed to participate in the project by logging in to the research website and completing the questionnaire.

## Results

### Preliminary analysis

All variables in the study were compared between the Polish and French samples. The results of the analysis are reported in S1 Table. Significant differences between the two countries were revealed in the following areas: the experience of a significant win in the environment (significantly more often in Poland); the preferred type of game (the lottery was chosen significantly more often in Poland); the amount spent on gambling (in Poland, they significantly more often included average amounts (second and third quartile); and, in France, the lowest and highest amounts (the first and fourth quartiles) and the frequency of gambling (significantly higher in the group of French gamblers).

### Significant wins and their naïve definitions among winners

When it comes to a significant win, 32.0% of the French sample reported having had a significant win experience (95% confidence interval [CI] [30.8%, 33.3%], *n* = 1,824); in the Polish group it was 38.7% (95% CI [37%, 40.4%], *n* = 1,216), and it was significantly more frequent than in France, χ^2^(1) = 39.61, *p* < .001. The nature of a significant win (one, multiple, series), experienced by the respondents differed significantly in both countries, χ^2^(2) = 64.13, *p* < .001. Numerous significant wins (37.1%, adjusted standardized residual (*e*) = 6.7) and a series of significant wins (5.1%, *e* = 3.6) were more often indicated by Polish respondents than French respondents, and only one significant win was experienced significantly more often by French gamblers (71.6%, *e* = 7.9). It is worth noting that the category of one significant win was most often indicated in both countries.

The next step was to analyze the meanings respondents from both countries ascribed to a “significant win.” Table 3 shows the respondents’ frequency with which respondents endorsed particular meanings and the differences between the two countries.

French gamblers considered significant wins more frequently than Polish gamblers as including a large amount of money and being added to the personal budget; also, the ones that followed a significant loss resulted from a low stake. In contrast, for Polish gamblers, a significant win was perceived more often as occurring in the context of losses and adverse events: it was used for the game and lost, it did not allow one to cover losses, it allowed the gambler to cover only a part of all losses, or it appeared under challenging life circumstances.

We also compared the two countries in terms of the amounts of wins the players considered significant. Because of the economic differences between Poland and France, we normalized these amounts in terms of both countries’ average monthly personal income [48]. Because of the discrepancy between the distributions of the amounts and normal distribution, we performed the comparison using a nonparametric Mann–Whitney *U* test. Significant differences were found (*U* = 1,345,059, *p* < .001). The median of normalized wins in Poland was higher (*Mdn* = 0.1659) than in France (*Mdn* = 0.0879).

### Prediction of a significant win experience

The next step of the analysis was to compare the predictors of experiencing significant winnings in France and Poland, which we carried out using logistic regression. Cross-cultural differences in prediction by individual factors were modeled through the interactions of these factors with the country variable. We performed the regression in blocks to incrementally analyze the prediction of groups of variables and their intercultural differentiation. In Block 1, sociodemographic variables related to the social status of the respondent were introduced: country, gender, age, education, household income, and debt. Block 2 contained the interactions of these variables with the country variable. We subsequently removed nonsignificant interactions. In Block 3, variables related to the social impact of gambling onset were introduced: the gambling onset year and the presence of a significant win in the respondent’s immediate environment. Block 4 contained interactions; only the significant ones were retained. Blocks 5 and 6 contained variables related to behavioral patterns of gambling, respectively: the type of gambling game, spending on gambling, frequency of gambling, and the interactions of these with the country. Block 7 and 8 included gambling motivations and their interactions with the country. Demographic variables related to the social and behavioral patterns have been previously recoded (the recoding description is in Table 2). The procedure results are included in S3 Table, and the summary of logistic regression models is given in Table 4. Based on this, the final common model was built after removing irrelevant interactions. Then, to assess the interaction effects, simple effects tests were carried out: Logistic analyses were carried out separately for both countries (see Table 5).

**Table 4:** Summary of logistic regression models built in the study.

Model	χ^2^	*df*	*p*	–2LL	Cox–Snell *R*^2^	Nagelkerke *R*^2^	Δχ^2^	Δ*df*	*p*
**Block 1**	247.99	7	< .001	10,328.1	.03	.04			
**Block 2**	255.29	9	< .001	10,320.8	.03	.04	255.29	9	< .001
**Block 3**	789.30	11	< .001	9,786.8	.09	.13	534.01	2	< .001
**Block 4**	798.09	12	< .001	9,778.0	.09	.13	8.80	1	.003
**Block 5**	1,244.50	16	< .001	9,331.6	.14	.19	446.41	4	< .001
**Block 6**	1,250.85	18	< .001	9,325.3	.14	.20	6.35	2	.042
**Block 7**	1,576.76	22	< .001	8,999.3	.18	.24	325.91	4	< .001
**Block 8**	1,596.01	24	< .001	8,980.1	.18	.24	19.25	2	< .001
**Final**	1,592.06	22	< .001	8,984.0	.18	.24			

*Note*. LL = log-likelihood.

**Table 5:** Logistic regression–the final common model and simple effects testing.

Predictor	Common model							France							Poland						
	*B*	*SE*	Wald	*df*	*p*	OR	95% CI	*B*	*SE*	Wald	*df*	*p*	OR	95% CI	*B*	*SE*	Wald	*df*	*p*	OR	95% CI
**Country (ref.: France)**	0.142	0.227	0.391	1	.532	1.152	[0.739, 1.797]														
**Gender (ref.: male)**	–0.060	0.055	1.192	1	.275	0.942	[0.846, 1.049]	–0.139	0.067	4.352	1	.037	0.870	[0.763, 0.992]	0.126	0.098	1.627	1	.202	1.134	[0.935, 1.375]
**Age (ref.: ≤34)**	–0.070	0.061	1.314	1	.252	0.932	[0.827, 1.051]	–0.042	0.077	0.301	1	.583	0.958	[0.824, 1.115]	–0.097	0.102	0.912	1	.340	0.907	[0.743, 1.108]
**Education (ref.: ≤SSC)**	0.095	0.053	3.184	1	.074	1.100	[0.991, 1.220]	0.113	0.066	2.938	1	.087	1.119	[0.984, 1.273]	0.039	0.092	0.182	1	.670	1.040	[0.868, 1.247]
**Household income (ref.: ≤MHI)**	0.028	0.055	0.251	1	.617	1.028	[0.923, 1.146]	0.042	0.068	0.382	1	.537	1.043	[0.913, 1.191]	–0.015	0.096	0.024	1	.877	0.985	[0.815, 1.190]
**Debts (ref.: None)**			53.893	2	< .001					33.749	2	< .001					21.289	2	< .001		
**Debts in the past**	0.411	0.057	52.599	1	< .001	1.509	[1.350, 1.687]	0.401	0.070	32.983	1	< .001	1.494	[1.303, 1.713]	0.448	0.098	20.788	1	< .001	1.565	[1.291, 1.897]
**Debts in the present**	0.294	0.083	12.711	1	< .001	1.342	[1.142, 1.578]	0.299	0.100	8.918	1	.003	1.348	[1.108, 1.641]	0.285	0.148	3.715	1	.054	1.330	[0.995, 1.777]
**Age at gambling onset (ref: ≤19)**	–0.463	0.067	48.494	1	< .001	0.629	[0.552, 0.717]	–0.459	0.068	45.850	1	< .001	0.632	[0.553, 0.722]	–0.123	0.094	1.712	1	.191	0.884	[0.735, 1.063]
**Significant win in an environment (ref.: No)**	0.855	0.057	228.805	1	< .001	2.351	[2.105, 2.627]	0.894	0.072	154.967	1	< .001	2.445	[2.124, 2.814]	0.789	0.092	73.661	1	< .001	2.201	[1.838, 2.636]
**Age at gambling onset × country**	0.350	0.111	9.927	1	.002	1.419	[1.141, 1.764]														
**Type of game (ref.: lottery)**	0.185	0.057	10.541	1	.001	1.203	[1.076, 1.344]	0.249	0.070	12.562	1	< .001	1.283	[1.118, 1.473]	0.026	0.099	0.072	1	.788	1.027	[0.847, 1.246]
**Spending on gambling (ref.: 1Q)**			43.617	2	< .001					41.942	2	< .001					38.233	2	< .001		
**Spending on gambling 2Q & 3Q**	0.225	0.081	7.762	1	.005	1.252	[1.069, 1.467]	0.230	0.082	7.867	1	.005	1.258	[1.072, 1.477]	0.373	0.115	10.492	1	.001	1.453	[1.159, 1.821]
**Spending on gambling 4Q**	0.631	0.098	41.322	1	< .001	1.880	[1.551, 2.279]	0.642	0.102	39.681	1	< .001	1.900	[1.556, 2.320]	0.986	0.160	37.925	1	< .001	2.680	[1.958, 3.667]
**Frequency of gambling (ref: Regularly, but less than once a week)**	0.399	0.063	40.288	1	< .001	1.490	[1.317, 1.685]	0.372	0.078	22.736	1	< .001	1.450	[1.245, 1.690]	0.472	0.108	19.067	1	< .001	1.603	[1.297, 1.981]
**Country × spending on gambling (ref.: 1Q)**			4.458	2	.108																
**Country × spending on gambling 2Q & 3Q**	0.165	0.138	1.439	1	.230	1.180	[0.901, 1.545]														
**Country × spending on gambling 4Q**	0.368	0.174	4.456	1	.035	1.444	[1.027, 2.032]														
**Enhancement motive**	0.369	0.052	49.650	1	< .001	1.446	[1.305, 1.603]	0.361	0.062	34.083	1	< .001	1.435	[1.271, 1.620]	0.374	0.100	13.889	1	< .001	1.453	[1.194, 1.769]
**Social motive**	0.084	0.067	1.588	1	.208	1.088	[0.954, 1.241]	0.054	0.085	0.396	1	.529	1.055	[0.893, 1.247]	0.126	0.109	1.334	1	.248	1.135	[0.916, 1.406]
**Coping motive**	0.365	0.080	20.712	1	< .001	1.440	[1.231, 1.686]	0.389	0.091	18.125	1	< .001	1.475	[1.233, 1.764]	0.611	0.120	25.850	1	< .001	1.843	[1.456, 2.333]
**Financial motive**	–0.047	0.040	1.401	1	.237	0.954	[0.882, 1.032]	–0.039	0.042	0.887	1	.346	0.962	[0.886, 1.043]	–0.316	0.064	24.199	1	< .001	0.729	[0.643, 0.827]
**Country × coping motive**	0.273	0.096	8.025	1	.005	1.314	[1.088, 1.587]														
**Country × financial motive**	–0.256	0.068	14.389	1	< .001	0.774	[0.678, 0.883]														
**Intercept**	–2.670	0.157	288.849	1	< .001	0.069		–2.699	0.170	250.854	1	< .001	0.067		–2.476	0.227	119.092	1	< .001	0.084	

*Note*. CI = confidence interval; MHI = mean household income; OR = odds ratio; PCG = pure-chance game; SSC+ = secondary school certificate.

The analysis showed that some predictors worked similarly in both countries, whereas the predictive power of others differed between countries. Moreover, among those that differed between the two cultures, some differed in effect size or the effect’s occurrence. However, most of the outcomes did not differ from country to country. These included: debt, a significant win in the environment, the type of game, the frequency of gambling, and the enhancement motive of gambling. Although significant in both cultures, two predictors had a greater effect in Poland: (1) gambling and (2) coping gambling motive. The effect of gambling onset age was significant only in France, and the financial gambling motive was significant only in Poland. Overall, there were more convergences than differences between the predictors of experiencing a significant win in Poland and France.

Having debts, both past (odds ratio [OR] = 1.51, *p* < .001) and present (OR = 1.34, *p* < .001), increased the likelihood of experiencing significant wins. Despite the country differences in defining a significant win mentioned above, in general, gamblers had a greater tendency to perceive a win as significant when they were in debt. Another factor with a positive effect is practicing a game of pure chance other than the lottery: This intensified the likelihood of declaring a significant win regardless of the sample (OR = 1.2, *p =* .001). Enhancement motivation to gamble worked similarly: In both countries, it intensified the likelihood of feeling a win was significant (OR = 1.45, *p* < .001).

A win of another person from the gambler’s immediate environment increased the chance of this experience (OR = 2.36, *p* < .001). Similarly, gambling frequency—playing at least once a week—significantly increased the likelihood of experiencing a significant win (OR = 1.50, *p* < .001).

As for the differences between countries in the prediction of experiencing a significant win, they concerned the effects of the following variables: age at gambling onset, spending on gambling, coping motive, and financial motive. Age at gambling initiation was a predictor of the experience of a significant win for French gamblers; as the age at onset increased (over 20 years), the likelihood of experiencing a significant decrease (OR = 0.63, *p* < .001). In contrast, in Poland the age at gambling onset was not explanatory (OR = 0.88, *p* = .191).

Another difference between both countries relates to gambling spending. Even though in both countries there was a positive relationship between the amount spent on gambling and the experience of a significant win, in Poland, the effect of the highest stakes was more substantial (OR = 2.68, *p* < .001) than in France (OR = 1.90, *p* < .001).

There were also differences in the motivation to gamble. In both countries, coping motivation was positively associated with the experience of a significant win, but this effect was more substantial in Poland (OR = 1.84, *p* < .001) than in France (OR = 1.48, *p* < .001). In turn, financial motivation reduced the likelihood of this experience in the group of Polish gamblers (OR = 0.73, *p* < .001); among French gamblers, this motivation was not a predictor of significant winning experience (OR = 0.96, *p* = .067).

## Discussion

Initial analyses of the differences between Poland and France demonstrated that players experienced a win in their close personal circle more often in Poland; in addition, lottery games were also more frequently chosen than other games of pure chance, and the stakes more often fell within average ranges. In France, we noted a greater frequency of gambling and occurrence of stakes in the low and high amounts ranges.

The results show that Polish gamblers often experienced a significant win over French gamblers. The definition of a significant win (no objective criterion, e.g., amount) we adopted means that we focused primarily on the subjective experience of gamblers. However, this understanding of winning seems more critical in its influence on further gambling. The total winnings may have different meanings for gamblers, depending on their material status. Overall, Polish players of pure chance games of chance more often claimed that they had had an important win. In addition, they stated that they had experienced many of these wins significantly more often than French gamblers, whereas French gamblers more often declared a single win than Polish gamblers.

It is interesting how gamblers from both countries defined a win they considered significant. Such an event was overwhelmingly positive for French gamblers and marked a robust emotional memory [54]. Therefore, the context in which the win occurs—the level of gambling practice or the uses the French gamblers will make with this win—validates the concept of a significant win. For Polish gamblers, a win was perceived significantly more often when it occurred in the context of losses and adverse events. The most apparent difference that emerged between gamblers concerns the overtones of such a win. In the case of French gamblers, it was accompanied by a halo of positive impressions; in the case of Polish gamblers, memories of adverse events. The results showed a more intense orientation of Polish gamblers toward financial wins than French ones. This fact may intensify Polish players’ tendency to complain about the game’s result because of the increased experience of losses, which is inevitable in gambling.

The results of other Polish nationwide surveys of gamblers have shown that the primary motivation for Polish players to engage in lotteries is financial [55]. However, qualitative research on older French adults has shown that this motivation is not essential [56]. Cultural differences in the perception of success may be another factor in interpreting our results, mainly the so-called “culture of complaining” [57]. Polish research drew attention to the culture of complaining that characterizes Polish society. For example, there is resistance among Poles against talking about successes because it is perceived negatively, whereas complaints dominate the discourse. Also, in Polish society successful people (politicians or business people) are perceived as less moral [57–59]. Interestingly, when relativized in terms of average monthly income, the significant wins were notably higher in the Polish sample than in the French one, confirming the Polish tendency to complain.

Logistic regression allowed us to develop the explanatory model common to both countries, including the following factors: having debts, age at gambling onset, significant win in a particular environment, type of game, spending on gambling, frequency of gambling, and motivation to gamble.

Being in debt may render any additional amount (of a win, no matter how high) perceived as significant, giving hope for another influx of cash obtained through gambling. Previous studies have demonstrated that indebtedness is strongly related to impulsive gambling and problem gambling and plays a mediating role between gambling and experiencing stress [60–62]. This result is consistent with the outcome concerning another explanatory factor—the frequency of gambling—which may be related to the level of indebtedness. According to the rules of gambling, the player loses money to the operator in the long term: The more intense the game (more frequent, lasting longer), the more intense the subjective sense of winning (unsystematic positive reinforcements necessary to continue the game) is but, at the same time, the higher the objective debt, as confirmed by research [55, 63, 64]. On the other hand, in the light of the cognitive approach, the more financial losses the player experiences, the more they try to justify their gambling, which may explain why they report significant wins more often [65]. Another factor that explains the claims of a significant win was experiencing a significant win by another person in their close personal circle. The players who stated they had had such an experience must have witnessed a vividly etched win in their memory because of its objective amount or the surrounding circumstances (e.g., the high intensity of emotions involved). In the light of cognitive psychology research, events accompanied by additional strong stimuli intensify the experience, which makes such events appear more attainable in the future and, therefore, more frequent [66, 67]. Playing pure chance gambling games other than lotteries (i.e., slot machines, roulette, scratch cards, and other casino games, except for poker and sports betting) also explained reporting a significant win. Lotteries are the most popular game in Poland and France [41, 42]. Playing the lottery usually involves low stakes and frequent small wins, with huge wins occurring more rarely [55]. It is interesting that although the ratio of the game expenditure and the wins reported by Polish players is similar in various gambling games (the expenses are almost twice the wins), it is the objective value of expenses—and, thus, wins—that is several times lower for lotteries, compared with other gambling games, in particular slot machines and casino games [55]. This may result in a poorer memory record of the lottery wins because of their subjectively lower value. Also, losing on slot machines is often disguised as a win (losses disguised as a win); that is, the actual win is smaller than the amount of money wagered, which may result in an overestimation of wins, including significant wins [68]. The research confirms the underestimation of losses on slot machines [61]. In addition, winning a casino or slot machine game is accompanied by audio-visual stimuli, which enhance the sensation of experiencing a win, even if it is a loss disguised as a win [67]. Here, it is worth mentioning that an important factor explaining a significant win was also the quantity of game expenditure: The higher it was, the greater the probability of experiencing a significant win, which may also result from the intensity of the game and the subjective impression of frequent wins.

The last, equally important factor in explaining significant wins in both countries was the enhancement motivation to gamble, that is, to experience positive emotions while gambling. One can expect that players who look for such experiences derive satisfaction from the uncertainty accompanying the game, and wins are an additional enhancement, providing further emotional stimulation regardless of the objective amount. Such motivation causes win to be experienced more intensely and thus described by players as significant. Interestingly, other studies have indicated that enhancement motivation is attributed to the players engaging in skill games [69, 70].

Another motive explaining a significant win experience was the coping motive, which exerted a more substantial effect on the Polish players but increased the probability of experiencing significant wins in both groups. This is another motive related to emotional management, except that, in this case, it reduces negative emotions. Gambling wins as a source of enjoyable experiences, temporarily reducing unpleasant sensations. In the case of players with such an orientation, each win, as something very desirable, is intensely experienced and probably perceived more strongly. It is interesting that the coping motivation is attributed to players choosing games of luck [69, 70]. The financial motivation, in turn, reduced the probability of experiencing a significant win, but only among Polish gamblers. This might mean that the Polish players oriented toward a financial win had higher expectations regarding wins, so small and medium amounts were less likely to mean “significant” to them. In contrast, in France, the financial value of the win seemed to weigh less in this definition. We confirmed this by analyzing the amounts defined as significant, which were higher for Polish players than for French players. The last factor separating Polish and French players in explaining a significant win was the win’s connection with the age of gambling initiation. In the French group, players under 20 years had more experience than older ones. In Poland, the age effect was not observed. This outcome can be explained by, for example, the popularity of games in particular age groups in France. For example, lotteries (games that do not generally result in a significant win) are the most popular among people over 35, and slot machines (one of the types of games that are connected to the significant win experience) are most popular among the youngest group of adults [71].

The following results draw attention as we summarize the importance of the factors explaining the experience of a significant win in both countries. First, there were no reverse patterns for both countries (positive factor relationship in one country and negative in another). Second, country-specific explanatory factors were observed. For Polish gamblers, this was the financial motivation; for French gamblers, the age at gambling onset. Other differences concerned the intensity of the effect (in the same direction) of specific factors: spending on gambling (higher in Poland) and coping motivation (higher in Poland).

## Limitations and conclusion

This study has some limitations. First, the representative sample of the French and Polish population, aged 18 to 64 in terms of distribution by sex and age, may have been biased because it was not random. Online surveys are generally nonprobabilistic—their sample is not controlled—so it is unknown who can answer them or who does answer them.

In addition, information about significant winnings, the gambler’s situation, and reactions are necessarily retrospective, which means that there are memorization biases (biases related to the player’s current situation, reconstruction of their past to trace a coherent history of their life).

The study’s results demonstrate the relevance of the chosen methodological approach, built on the concept of a significant win based on the gambler’s perception without imposing any predefined criteria. The proposed definition has made it possible for us to consider better all subjective perceptions of winning situations that may affect gambler’s career and the possible difficulties they may encounter. Overall, the study provides relevant lessons that could be considered in prevention and harm reduction strategies. A significant win was defined in terms of monetary value, time, context, use, or gambling practice. Our research results emphasize the need for preventive actions devoted to shaping the awareness of random mechanisms of winning in gambling, as well as minimizing the effects of modeling in gambling activity, especially among young people. Furthermore, it seems justified to investigate further investigations of the importance of a significant win in developing an addiction to gambling seem justified in the context of the results obtained. Also, an in-depth study of the importance of having players in close vicinity seems essential. Finally, this research revealed some intercultural differences in the perceptions of a win between players from Poland and France. Cultural factors can therefore play a vital role in explaining engaging in gambling.

## Supporting information

S1 Table Comparison of gambling-related variables between the Polish and French samples. (XLSX)

S2 Table Testing of GMQ–F invariance between the Polish and French samples. (XLSX)

S3 Table Logistic regression: Results of the block procedure. (XLSX)

S4 Table Logistic regression: The final common model and simple effects testing. (XLSX)
